# Endomyocardial biopsy in the clinical context: current indications and challenging scenarios

**DOI:** 10.1007/s10741-022-10247-5

**Published:** 2022-05-14

**Authors:** Aldostefano Porcari, Chiara Baggio, Enrico Fabris, Marco Merlo, Rossana Bussani, Andrea Perkan, Gianfranco Sinagra

**Affiliations:** 1 Centre for Diagnosis and Treatment of Cardiomyopathies, Cardiovascular Department, Azienda Sanitaria Universitaria Giuliano-Isontina (ASUGI), University of Trieste, Via P. Valdoni 7, 34100 Trieste, Italy; 2 Cardiothoracic Department, Center for Diagnosis and Treatment of Cardiomyopathies, Institute of Pathological Anatomy and Histology, Azienda Sanitaria Universitaria Giuliano-Isontina, University of Trieste, Trieste, Italy

**Keywords:** Endomyocardial biopsy, Diagnosis, Cardiomyopathies, Cardiovascular imaging, Clinical practice

## Abstract

Endomyocardial biopsy (EMB) is an invasive procedure originally developed for the monitoring of heart transplant rejection. Over the year, this procedure has gained a fundamental complementary role in the diagnostic work-up of several cardiac disorders, including cardiomyopathies, myocarditis, drug-related cardiotoxicity, amyloidosis, other infiltrative and storage disorders, and cardiac tumours. Major advances in EMB equipment and techniques for histological analysis have significantly improved diagnostic accuracy of EMB. In recent years, advanced imaging modalities such as echocardiography with three-dimensional and myocardial strain analysis, cardiac magnetic resonance and bone scintigraphy have transformed the non-invasive approach to diagnosis and prognostic stratification of several cardiac diseases. Therefore, it emerges the need to re-define the current role of EMB for diagnostic work-up and management of cardiovascular diseases. The aim of this review is to summarize current knowledge on EMB in light of the most recent evidences and to discuss current indications, including challenging scenarios encountered in clinical practice.

**Supplementary information:**

The online version contains supplementary material available at 10.1007/s10741-022-10247-5.

## Introduction

Endomyocardial biopsy (EMB) is an invasive procedure developed in 1960s initially for the early diagnosis and monitoring of heart transplant (HTx) rejection [1]. Since then, this technique has become an important tool for the diagnosis and evaluation of different cardiac disorders such as cardiomyopathies, myocarditis, infiltrative and storage diseases, and cardiac tumours. EMB incremental diagnostic, prognostic and therapeutic value has led to a wide diffusion of this technique over the years in different settings. Nevertheless, a significant heterogeneity exists in EMB use in clinical practice, probably due to its low sensitivity in specific scenarios, the development of non-invasive diagnostic techniques, the non-negligible risk of major complications especially in non-experienced centres and the limited availability of skilled cardio-pathologists for the interpretation of histological findings, outside referral centres [2].

In recent years, the use of advanced imaging modalities, as echocardiography with three-dimensional (3D) and myocardial strain analysis, cardiac magnetic resonance (CMR) and positron emission tomography (PET), has revolutionized the non-invasive approach to diagnosis and prognostic stratification of several cardiac diseases, resulting in an accurate selection of cases in which EMB may carry important both diagnostic and prognostic information (Table 1). Indeed, along with a full morphological and functional cardiac assessment, CMR imaging can provide a detailed characterization of the cardiac muscle composition, although it may not be feasible in patients with specific features (i.e. arrhythmias, poor breath-holding, non-conditioned cardiac devices) [3]. A remarkable example is the possibility to reach a non-invasive diagnosis of acute myocarditis (AM), according to the Lake Louise Criteria published in 2009 [4], updated in 2018 [5]. Furthermore, another promising tool for clinical practice is the measurement of myocardial strain, which can reveal subtle systolic dysfunction in patients with clinically suspected AM or anthracycline-induced cardiomyopathy presenting with apparently normal left ventricular ejection fraction (LVEF) [6, 7]. In addition, PET with 18F-fluorodeoxyglucose (18F-FDG) uptake could help to identify the involvement of the heart in cardiac sarcoidosis (CS) and to monitor treatment response [8]. Over the years, major technical advances in EMB, tissue processing and analysis, such as molecular diagnostics, proteomics and electron microscopy, increased the accuracy of this procedure and reduced the risk of major complications.

**Table 1:** Sensitivity and specificity of EMB, CMR, scintigraphy, and PET selected cardiac disease

**Disease**	**Technique**	**Finding**	**Sensitivity**	**Specificity**
Myocarditis	CMR [4, 5, 45]	EGE suggests hyperaemia and capillary leak. LGE detects cell necrosis and fibrosis. T2-weighted imaging and T2 mapping identify myocardial oedema	67%	91%
	EMB [16]	Histologic and immunohistochemical criteria	Diagnostic accuracy of: • 79.3% in LV-RV biopsy • 67.3% in LV or RV biopsy	
Sarcoidosis	CMR [9]		93%	85%
	PET [46, 47]	Active inflammation and scar	89%	78%
	EMB [48]	Non-caseating granulomas	< 20–25%	/
Amyloidosis	CMR [49, 50]	Unable to differentiate AL from ATTR cardiac amyloidosis	86%	92%
	Bone tracer scintigraphy [27]	Myocardial uptake in ATTR-CA	99%	86%
	PET [51]	Discriminating CA, especially AL, from controls	94%	93%
	EMB [27]	Amyloid deposition	≈100% if ≥ 4 samples collected	≈100% if ≥ 4 samples collected

*AL* light chain amyloidosis, *ATTR* transthyretin amyloidosis, *CA* cardiac amyloidosis, *CMR* cardiac magnetic resonance, *EGE* early gadolinium enhancement, *EMB* endomyocardial biopsy, *LGE* late gadolinium enhancement, *LV* left ventricular, *PET* positron emission tomography, *RV* right ventricular

Therefore, it emerges the need to re-define the current role of EMB for diagnostic work-up and management of cardiovascular diseases, a task recently pursued by an international “Position statement on endomyocardial biopsy” [9]. The aim of this review is to summarize current knowledge on EMB in light of the most recent evidences and discuss current indications, including challenging scenarios encountered in clinical practice.

## The current role of EMB

Providing essential information on myocardial histology, immunohistochemistry and molecular structure, EMB represents the gold-standard technique to reach a definite and etiological diagnosis in different cardiac disorders, to improve patients’ stratification and guide treatment options (Table 2) [10, 11]. Emerging imaging modalities (3D echocardyography, CMR, PET, electro anatomic voltage mapping) guiding cardiac sampling and the implementation of immunohistochemistry and polymerase chain reaction (PCR) to standard histologic evaluation have enhanced EMB diagnostic accuracy [12]. However, a significant variability in EMB diagnostic yield is conferred by the specific pattern of myocardial involvement (i.e. focal vs diffuse tissue) and centre’s expertise in samples’ collection, processing, analysis and interpretation.

**Table 2:** Progressive evolution in the recommendations of EMB over time

**Study**	**Type of document and written associations**	**Recommendations**
Cooper et al. [11]	Scientific Statement from: AHA, ACC, ESC	**- 14 clinical scenarios** in which EMB had a diagnostic, prognostic, and therapeutic value:
		1) New-onset HF of 2 weeks’ duration with hemodynamic compromise
		2) New-onset HF of 2-week to 3-month duration with a dilated LV and new ventricular arrhythmias, high AV block, or failure to respond to usual care within 1 to 2 weeks
		3) HF of 3 months’ duration with a dilated LV and new ventricular arrhythmias or high-degree heart block, or failure to respond to usual care within 1 to 2 weeks
		4) HF with a DCM of any duration associated with suspected allergic reaction and/or eosinophilia
		5) HF with suspected anthracycline cardiomyopathy
		6) HF with unexplained restrictive cardiomyopathy
		7) Suspected cardiac tumours
		8) Unexplained cardiomyopathy in childre
		9) New-onset HF of 2-week to 3-month duration associated with a dilated LV, without new ventricular arrhythmias or AV block, that responds to usual care
		10) HF of 3-month duration with a dilated LV, without new ventricular arrhythmias or AV block, that responds to usual care
		11) HF with unexplained HCM
		12) Suspected ARVD/C
		13) Unexplained ventricular arrhythmias 14) Unexplained atrial fibrillation
Jessup et al. [52]	Guidelines for heart failure from: ACC, AHA	**- Indications for EMB:**
		Monitor cardiac transplant rejection status
		Diagnose unexplained cardiomyopathies
		Suspected myocarditis
		Suspected infiltrative cardiomyopathy
		Diagnose cardiac tumours
		Detect suspected anthracycline toxicity
		Use in research
Seferović et al. [9]	Consensus document of the trilateral cooperation between: ESC, HFSA, HFA, JHFS	**- Updated Indications for EMB** (9 scenarios, see Table 4) - Recommended schedule for HTx rejection surveillance EMB
McDonagh et al. [25]	Guidelines for the diagnosis and treatment of acute and chronic heart failure from: ESC	- EMB remains the gold-standard investigation for the identification of cardiac inflammation - It may confirm the diagnosis of autoimmune disease in patients with DCM and suspected giant cell myocarditis, eosinophilic myocarditis, vasculitis and sarcoidosis - It may also help for the diagnosis of storage diseases, including amyloid or Fabry disease, if imaging or genetic testing does not provide a definitive diagnosis - It might be considered also in HCM if genetic or acquired causes cannot be identified - The risks and benefits of EBM should be evaluated and this procedure should be reserved for specific situations where its results may affect treatment

*ACC* American College of Cardiology, *AHA* American Heart Association, *ARVD* arrhythmogenic right ventricular dysplasia, *AV* atrioventricular, *DCM* dilated cardiomyopathy, *EMB* endomyocardial biopsy, *ESC* European Society of Cardiology, *HCM* hypertrophic cardiomyopathy, *HF* heart failure, *HFA* Heart Failure Association, *HFSA* Heart Failure Society of America, *HTx* heart transplant, JHFS Japanese Heart Failure Society, *LV* left ventricular

EMB is a cornerstone in the management of patients with unexplained acute heart failure (HF) with haemodynamic compromise or ventricular arrhythmias/conduction disorders of unknown aetiology, clinically suspected AM, CS, storage and infiltrative diseases, cardiac masses and monitoring of HTx rejection status [10, 11].

Aside diagnostic information, viral search by PCR, reverse transcription (RT)-PCR and direct sequencing analysis on cardiac specimens have also therapeutic implications, as in the evaluation of possible candidates to immunosuppressive therapy [13]. While immunosuppression lacks conclusive prognostic evidence in virus-negative lymphocytic myocarditis, it is a recognized effective treatment option for other inflammatory cardiomyopathies, such as giant cell myocarditis (GCM), eosinophilic necrotizing myocarditis (ENM) and CS, which are associated with a poor outcome [13, 14]. Finally, during COVID-19 pandemic, although rarely indicated in suspected AM related to SARS-Cov-2, EMB has been used mainly for research purposes. In this setting, histopathological finings revealed an increased macrophage and lymphocytic tissue infiltration in the heart, without evidence of viral presence within cardiomyocytes [15].

## EMB: the procedural steps in the lab and potential issues

**Fig. 1:**
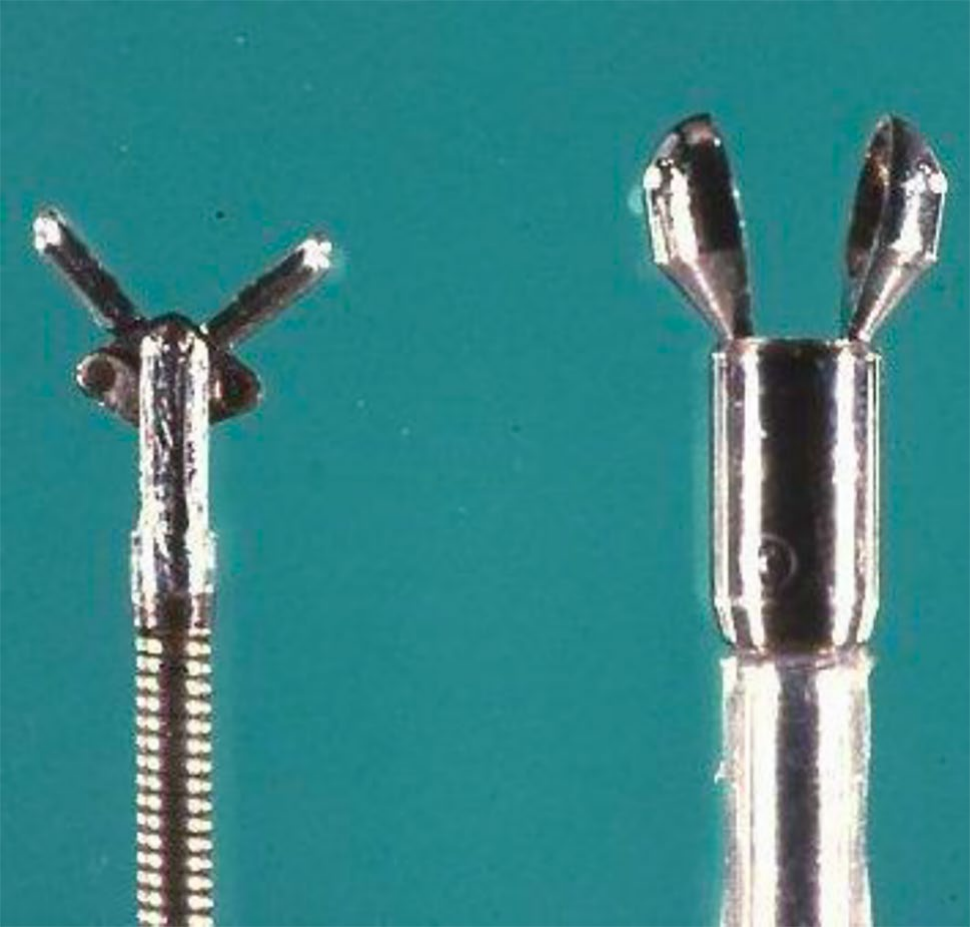
Old generation bioptomes for endomyocardial biopsy

The first technique of EMB using a bioptome designed for transvascular approach (Konno-Sakakibara bioptome) was reported in the early 1960 in Japan [1]. Over the last 60 years, EMB technique has been refined and the procedure has gained worldwide acceptance (Fig. 1). However, safety remains a concern when performing EMB; indeed, although rare, major complications (including cardiac tamponade necessitating pericardiocentesis, thromboembolism, severe arrhythmias/atrioventricular block, valvular trauma) occur in about 1% of cases [16, 17]. Haemodynamically unstable patients and those with large ventricles with thin walls may be at a higher risk of cardiac perforation, which is more frequently observed with right ventricle (RV) than with left ventricle (LV) EMB (Table 3). Of note, LV EMB is associated with a higher risk of stroke or systemic embolism. Therefore, when posing indications to EMB, the risk/benefit balance should be accurately evaluated and the procedure should be performed in high-volume centres with specific expertise to minimize the risk of such complications [2].

**Table 3:** Comparison of minor and major complication rates in LV EMB and RV EMB according to different studies and centres

**Study (state, years)**	**Procedural complications**	**LV EMB**	**RV EMB**
Göbel et al. [20] (Germany, 2013–2018)		*n* = 461	*n* = 53
	**Bradiarrhythmias**		
	Permanent AV block	1 (0,2%)	1 (2%)
	Transient AV block	0 (0%)	1 (2%)
	**Tachyarrhythmias**		
	Atrial fibrillation	Not reported	Not reported
	Non-sustained VT	2 (0,4%)	0 (0%)
	Ventricular fibrillation	0 (0%)	0 (0%)
	**Vascular complications**	0 (0%)	0 (0%)
	**Pneumotorax**	Not reported	Not reported
	**Infections**	Not reported	Not reported
	**Pericardial complications/perforations**		
	Pericardial effusion*	46 (10%)	4 (8,5%)
	Cardiac tamponade	0 (0%)	0 (0%)
	**Stroke/TIA**	3 (0,6%)	0 (0%)
	**Death**	0 (0%)	0 (0%)
Chimenti and Frustaci [17] (USA, 1983–2010)		*n* = 3549	*n* = 3068
	**Bradiarrhythmias**		
	Permanent AV block	0 (0%)	0 (0%)
	Transient AV block	0 (0%)	2 (0,06%)
	**Tachyarrhythmias**		
	Atrial fibrillation	Not reported	Not reported
	Non-sustained VT	6 (0,16%)	4 (0,13%)
	Ventricular fibrillation	Not reported	Not reported
	**Vascular complications**	17 (0,48%)	6 (0,19%)
	**Pneumotorax**	Not reported	Not reported
	**Infections**	Not reported	Not reported
	**Pericardial complications/perforations**		
	Pericardial effusion	1 (0,03%)	5 (0,16%)
	Cardiac tamponade	3 (0,08%)	9 (0,29%)
	**Stroke/TIA**	8 (0,22%)	0 (0%)
	**Death**	0 (0%)	0 (0%)
Yilmaz et al. [16] (USA, 2006–2008)		*n* = 622	*n* = 490
	**Bradiarrhythmias**		
	Permanent AV block	Not reported	Not reported
	Transient AV block	0 (0%)	1 (0,2%)
	**Tachyarrhythmias**		
	Atrial fibrillation	Not reported	Not reported
	Non-sustained VT	3 (0,5%)	3 (0,6%)
	Ventricular fibrillation	Not reported	Not reported
	**Vascular complications**	Not reported	Not reported
	**Pneumotorax**	Not reported	Not reported
	**Infections**	Not reported	Not reported
	Pericardial complications/perforations*		
	Pericardial effusion	14 (2,3%)	14 (2,9%)
	Cardiac tamponade	2 (0,3%)	4 (0,8%)
	**Stroke/TIA**	2 (0,3%)	0 (0%)
	**Death**	0 (0%)	0 (0%)

*AV* atrioventricular, *EMB* endomyocardial biopsy, *LV* left ventricular, *RV* right ventricular, *TIA* transient ischemic attack, *VT* ventricular tachycardia. *Pericardial effusion was a transient phenomenon and no perforation was documented

RV EMB is performed by jugular, femoral or brachial veins. Transradial artery access is emerging as an alternative access route for LV EMB [18, 19], non‐inferior to transfemoral artery access in terms of major complications [20] and possibly leading to fewer access-site bleedings compared to the femoral access [21]. However, LV EMB is still usually performed by femoral arteries. In LV EMB, intravenous heparin is given to reach an ACT > 200 s to reduce the risk of embolism. From femoral access, long sheath technique is predominantly used for semi-flexible bioptomes also to avoid repeated exposure of the valve leaflets to the bioptome. The long sheath is introduced in the ventricle over a pigtail catheter which advances over a 0.035 wire under fluoroscopy guidance. Then, the mid LV cavity position of the tip of the sheath is confirmed in right and left anterior oblique projection in order to avoid the apex and to be far from valvular apparatus. At this stage, performing a ventriculography (Video 1) can facilitate the positioning of the catheter and additional angiographic views can be used for specific site of sample collection. Pigtail catheter is then removed and the bioptome is advanced into the LV. The forceps should be already in the “opened” position inside the distal segment of the long sheath and have to remain open until contact with the ventricular wall. The bioptome forceps are closed when a slight resistance is sensed by the operator; ventricular beat or non-sustained ventricular tachycardia is common while the bioptome is in contact with the myocardium. The bioptome is then removed from the sheath and the sheath is aspirated and flushed to prevent air or tissue embolism. Once the patient’s hemodynamic stability has been ascertained, heparin is antagonized with protamine sulphate and the introducer removed by closure devices. The general advice about steering the bioptome holds true for both LV and RV EBM. Of note, in RV EMB, samples are usually taken from different sites of the interventricular septum (Videos 2 and 3) to reduce the risk of perforation.

A successful procedure should provide at least ≥ 5 samples for histological evaluation, immunohistochemistry and viral PCR analyses [11, 22]. The diagnostic yield of EMB may be optimized when samples from both ventricles are available [16]; however, LV EMB appears diagnostically more informative than RV EMB [17] in patients with clinically uninvolved RV. The best approach should be identified based on an accurate clinical query: LV biopsy is preferred in suspected AM with primary LV involvement and CS. While the diagnostic accuracy of EMB is high in diffuse cardiac diseases, such as CA, collection of specimens from multiple cardiac sites should be considered in focal diseases as in CS [23]. Of note, increasing the number of collected samples is paralleled by a higher risk of complications [24]. Pre-operative third-level imaging techniques as CMR and 18F-FD PET may identify the sites with myocardial fibrosis (LGE and T1 mapping), cardiac edema (T2 mapping), and inflammation where the probability of an informative EMB result is higher. Moreover, the use of intra-procedural electroanatomic mapping may detect ventricular segments with fragmented or low voltages [5] which may indicate the most diseased area for sample collection [8]. Further studies are needed to evaluate the ability of imaging techniques and electroanatomic mapping to guide EMB and improve the safety and the diagnostic yield of the procedure.

## Clinical indications to EMB

Clinical indications to EMB have been based on empirical decisions and expert opinions, often heterogeneous worldwide and dynamic over time, mostly due to the invasive nature of this technique and the lack of specific clinical trials and guidelines (Table 2). Previously published international Scientific Statements [10, 11] did not result in a standardized use of EMB in clinical practice. Currently, in the latest guidelines on the diagnosis and treatment of HF from the European Society of Cardiology (ESC), EMB is indicated with a class IIa recommendation in the context of rapidly progressive HF despite standard therapy, when there is a probability of a specific diagnosis requiring specific treatments, which can be confirmed only in myocardial samples [25].

Furthermore, the recent position Statement by Seferovic et al. [9] identified with a standardized approach 9 specific clinical scenarios in which EMB should be considered to reach the final diagnosis and to guide decision-making (Table 4).

**Table 4:** Clinical indications and contraindications for endomyocardial biopsy

**Indications**	**Contraindications**	
	Absolute	Relative
Suspected fulminant/acute myocarditis with acute HF and/or rhythm disorders or suspected myocarditis in haemodynamically stable patients	Intracardiac thrombus	Infective endocarditis
DCM with new-onset HF and LV dysfunction, non-responsive to standard medical therapy	Severe aortic, pulmonary or tricuspid stenosis	Active infection
Unexplained hypertrophic or restrictive myocarditis	Aortic and tricuspid mechanical prosthesis	Cerebrovascular accident/TIA < 1 month before
Unexplained ventricular arrhythmias, high-degree atrioventricular block and/or syncope	Ventricular aneurysm	Uncontrolled hypertension
Autoimmune disorders with progressive HF refractory to treatment		Active bleeding
Suspected ICI-mediated cardiotoxicity		Pregnancy
MINOCA/Takotsubo syndrome with progressive HF and LV dysfunction		Contrast media hypersensitivity
Cardiac tumours		Thin ventricular wall
HTx rejection status monitoring		Coagulopathy
		Uncooperative patients

*DCM* dilated cardiomyopathy, *HF* heart failure, *HTx* heart transplant, *ICI* immune checkpoint inhibitors, *LV* left ventricular, *MINOCA* myocardial infarction without obstructive coronary artery disease, *TIA* transitory ischemic attack

### Use of EMB in different clinical settings

EMB covers a fundamental role in suspected AM presenting with cardiogenic shock or acute HF with ventricular dysfunction and/or rhythm disorders. DCM with recent onset and progressive HF, unresponsive to standard treatment, or with new-onset unexplained sustained ventricular arrhythmias as well as high-degree atrioventricular blocks is a setting where EMB is considered useful. These two latter presentations in the context of an autoimmune disorder represent a scenario in which EMB may confirm the presence of an autoimmune myocarditis (i.e. GCM, CS) or vasculitis in patients with unexplained DCM [9]. In these contexts, EMB results have a crucial role in orienting immunosuppressive treatment.

EBM may also be considered in unexplained cardiomyopathies with hypertrophic or restrictive phenotype and inconclusive non-invasive results [26]. In detail, EMB can be useful in patients with a clinical context of high suspicion for HCM phenocopies such as CA. EMB is the gold standard for the diagnosis of CA with nearly 100% sensitivity and specificity if specimens are collected from > 4 multiple sites and tested for amyloid deposits by Congo red staining [27]. In this setting, EMB or extracardiac biopsy is recommended to confirm light chain (AL) CA in patients with suggestive non-invasive findings and evidence of monoclonal proteins [26]. Conversely, a final diagnosis of transthyretin-related (ATTR) CA can be achieved noninvasively in patients with grades 2–3 cardiac uptake at bone tracers scintigraphy and absence of monoclonal proteins on serum and urine tests [28]. Finally, EMB may be considered in the work-up of patients with suspected cancer therapy cardiotoxicity, in particular mediated by immune checkpoint inhibitors (ICI), and for the characterization of cardiac masses without high embolic risk [9]. In all the aforesaid settings, EMB is expected to be highly informative towards ongoing mechanisms of cardiac damage, throughout both the ultrastructural characterization of cardiac tissue and inflammatory infiltrates, and the detection of viral presence, toxic injuries and metabolic disorders [25].

## EMB use in selected controversial scenarios

It should be always considered that EMB is an invasive procedure to be used after careful consideration of risks and benefits [24]. EMB has some limitations [2] : (a) its accuracy is not 100% and inconclusive results are possible in clinical practice, (b) it is associated with a modest, but still relevant, risk of potential major procedural complications, (c) the presence of mild myocardial histological changes is not always clinically relevant and does not address a specific therapeutic approach, and (d) it requires cardiac pathologists with experience in the interpretation of histological findings. Finally, some absolute contraindications to EMB should be considered such as the presence of intracardiac thrombus, ventricular aneurysm, severe tricuspid, pulmonary or aortic stenosis and tricuspid or aortic prosthesis [9].

A careful candidate selection with a stepwise and comprehensive approach is recommended in challenging scenarios (Table 5). Of note, this approach to EMB indication is supported by the 2021 guidelines for the diagnosis and treatment of acute and chronic HF from the ESC [25].

**Table 5:** Endomyocardial biopsy use in challenging clinical scenarios

**Clinical scenarios**	**Key findings**	**Possible histological diagnosis**
Hemodynamically stable non-ischemic DCM not improving after ≥ 3 months of optimal medical therapy	• Family history of CMP or juvenile SCD • Persistently or relapsing increase in serum troponin • Skin abnormalities • Frequent ventricular ectopy or arrhythmias • Myopathy or syndromic features • Chemotherapy	• Myocarditis • Haemochromatosis • Undetermined CMP
Haemodynamically stable patients with clinically suspected AM and normal LVEF	• Persistently or relapsing increase in serum troponin • Development of LV dysfunction • Frequent ventricular ectopy or arrhythmias • Known extracardiac sarcoidosis • Systemic autoimmune disorders	• Lymphocytic myocarditis • GCM • Cardiac sarcoidosis • ENM • Chronic myocarditis
Hemodynamically stable patients with HF and unexplained cardiac hypertrophy	• Family history of CMP or juvenile SCD • Severe cardiac hypertrophy (i.e. > 30 mm) • Stroke or TIA (especially < 40 years) • Renal insufficiency (especially < 40 years) • CTS or polyneuropathy • QRS voltage/LV mass discrepancy • Pericardial effusion • Skin abnormalities • Vitreous abnormalities	• HCM • Fabry disease • Danon disease • PRKAG2 disease • Cardiac amyloidosis
Restrictive cardiomyopathy	• CTS or polyneuropathy • QRS voltage/LV mass discrepancy • Pericardial effusion • Vitreous abnormalities • Skin abnormalities • New-onset diabetes • Anaemia with serum ferritin > 300 ng/mL and transferrin saturation > 55%	• Cardiac amyloidosis • Haemochromatosis • Undetermined CMP
Cardiac mass	• Fever or increased inflammatory markers • Positive blood/urine culture • Atrial roof origin • Ventricular localization • Inconclusive non-invasive assessment	• Vegetation • Primary or secondary cardiac tumour

*AM* acute myocarditis, *CMP* cardiomyopathy, *CTS* carpal tunnel syndrome, *DCM* dilated cardiomyopathy, *ENM* eosinophilic necrotizing myocarditis, *GCM* giant cell myocarditis, *HCM* hypertrophic cardiomyopathy, *HF* heart failure, *LV* left ventricular, *LVEF* left ventricular ejection fraction, *SCD* sudden cardiac death, *TIA* transient ischemic attack

### Non-ischemic DCM and clinically suspected myocarditis

**Fig. 2:**
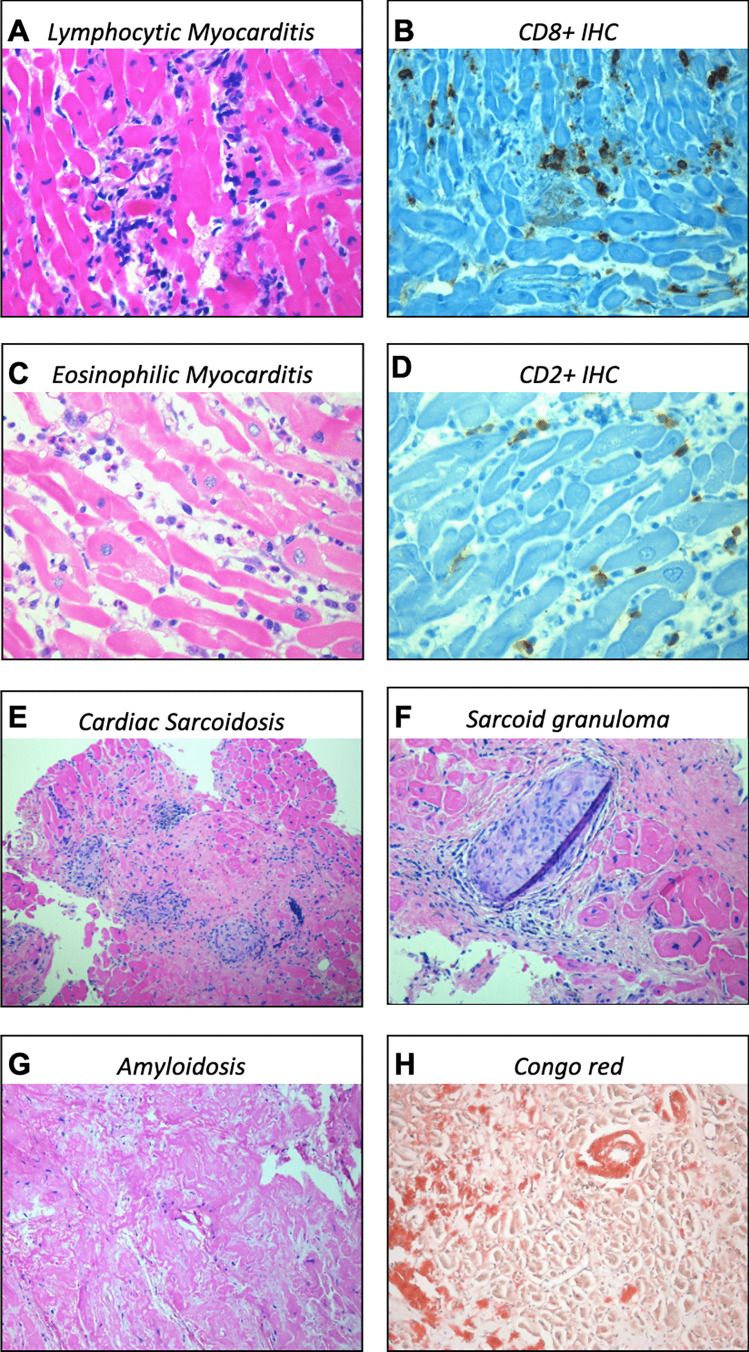
Possible histopathological findings in cardiac diseases. **A** and **B** Lymphocytic myocarditis with intense inflammatory infiltrates in the myocardium; **C** and **D** eosinophilic myocarditis with eosinophilic cells during active degranulation; **E** and **F** cardiac sarcoidosis with inflammatory infiltrates and modest myocardial fibrosis and the typical non-caseating sarcoid granuloma; **G** and **H** cardiac amyloidosis with vascular and interstitial deposition on Congo red staining. Legend: IHC, Immunohistochemistry

Non-ischemic DCM represents a particularly complex setting due to polymorphic clinical presentation and evolution [24]. Patients presenting with hemodynamically stable non-ischemic DCM are a challenging scenario where EMB might be essential to make the diagnosis and guide therapy [25] when no clinical improvement is seen after at least 3 months of optimal medical treatment, especially in absence of severe LV remodelling [29]. Among the several causes that may underlie this clinical presentation, AM is a reversible and treatable condition [30]. Accurate multiparametric non-invasive evaluation can strengthen the clinical suspicion. However, the value of EMB lies in the ability to orient diagnosis and immunomodulation strategies based on histopathological and immunohistochemical results, combined with the evaluation of viral presence in the heart via PCR analysis [12, 13]. In detail, histological evaluation provides key information on the presence, type and degree of inflammatory cells (i.e. lymphocytic vs non-lymphocytic myocarditis), presence of viruses, myocardial fibrosis or changes in myocardial architecture consistent with a cardiomyopathy substrate [13, 22]. In lymphocytic myocarditis, viral presence detected by PCR analysis on myocardial samples is a contraindication to immunosuppression, although PVB-19 with a low replicative activity (< 250–500 copies/μg) represents a condition where immunosuppression require further research in controlled clinical trials (Fig. 2) [13, 22].

Haemodynamically stable patients with normal LVEF and clinically suspected AM are another challenging scenario where EMB should be considered to reach a final diagnosis and guide therapy if persistently or relapsing increased serum troponin values, deterioration of systolic function or frequent ventricular arrhythmias are present.

CS is another challenging scenario where the patchy myocardial involvement makes it difficult to find the pathognomonic non-caseating granulomas. In case of strong clinical suspicion and consistent non-invasive tests (i.e. PET), a negative EMB result should not discourage clinicians to pursue a final diagnosis, also with a repeated EMB [13].

### Hypertrophic cardiomyopathies and phenocopies

EMB might be indicated to reach a final diagnosis in patients presenting with HF and ventricular hypertrophy. Patients’ age at clinical onset is relevant as the incidence of phenocopies (i.e. CA) increases with ageing [31], while HCM, Danon disease and Anderson-Fabry disease are more frequently encountered in young adults [32]. Specific extracardiac findings such as renal insufficiency and neurological issues might be found in Anderson-Fabry disease, while bilateral carpal tunnel syndrome (CTS) [33] and macroglossia should raise the suspicion of CA [28]. Extreme ventricular thickening (i.e. > 30 mm) with significantly increased QRS voltages in very young patients might suggest the presence of Danon disease [34]. Infiltrative diseases (i.e. CA) expand the extracellular space and can present with low QRS voltages, while storage diseases (i.e. Pompe disease, PRKAG2 disease) are characterized by normal to high QRS voltages [35]. The presence of discrepancies between the degree of ventricular hypertrophy at echocardiography and the QRS voltage on surface ECG might suggest an infiltrative disease [35]. Conversely, a family history of HCM or sudden death at a young age, significant cardiac hypertrophy with an asymmetrical pattern, especially involving the interventricular septum or the apex, and the identification of a genetic mutation in sarcomeric proteins suggest the presence of HCM. CMR can provide important information to differentiate HCM from phenocopies [3], but a final diagnosis can be made only by histological analysis in specific settings.

### Restrictive cardiomyopathies

EMB might be required in patients presenting with restrictive cardiomyopathy with controversial non-invasive findings to reach a final diagnosis (i.e. endomyocardial fibrosis, CA, hemochromatosis). Patients with iron overload cardiomyopathy (IOC) might present with restrictive phenotype on a background of acquired anaemia requiring multiple transfusions or development of diabetes mellitus with typical skin pigmentation. The diagnosis is supported by the presence of serum ferritin > 300 ng/mL with transferrin saturation > 55% and cardiac siderosis (cardiac T2* < 20 ms on CMR scan) [36]. However, in case of inconclusive non-invasive tests, cardiac samples analysis with Perls’ Prussian blue stain can provide histological confirmation of myocardial iron deposition [36].

### Cardiac masses

Patients with incidental detection of cardiac masses require accurate differential diagnosis between tumour, vegetation, calcification and thrombus [37]. The presence of fever, increased white blood cells count and PCR serum levels, signs of organ infection (i.e. pneumonia), cardiac devices, native or prosthetic valve disease and known neoplasia increase the risk of infective or non-infective endocarditis. Fatigue, anaemia and progressive weight loss might suggest the presence of a neoplasm. Contrast CT and CMR play a key role in characterization of the cardiac mass (i.e. CMR in lipomas) and in pre-procedural planning, while scintigraphy with 99-Tc-labelled leukocytes can reveal sites of active inflammation and infection [37]. Size and location of the mass in the heart are relevant: myxomas are found predominantly in the left atrium, lipomas tend to occur in right atrium or in the left ventricle, and fibroma and rhabdomyomas are mostly located in the ventricle. Some masses might show peculiar features such as myxomas, which can cause dynamic mitral valve obstruction leading to syncope, pulmonary edema and embolic manifestations [37]. Of note, EMB, particularly LV EMB, is not indicated in case of intracardiac friable masses with high embolic potential such as left-sided tumours or typical cardiac myxomas [9]. In those cases, a patient’s tailored approach is essential and surgical removal can be preferred over EMB, according to patients’ overall conditions.

### HTx rejection

EMB remains the gold standard for the diagnosis and monitoring of HTx rejection status. An International Society for Heart and Lung Transplantation (ISHLT) classification of postcardiac transplant cellular rejection was developed in 1990 and revised in 2004 [38], facilitating the standardization and management of graft rejection. However, poor inter-pathologists agreement in grading rejection has been a concern, as demonstrated by the data from the CARGO II study [39]. Standardization in diagnosis is still a challenge. Automated computational-image analysis and molecular diagnostics are promising tools to improve the precision and accuracy in the disease pathological grading and classification [10].

Another debated issue is the optimal timing and frequency of routine surveillance EMB (rsEMB) after HTx, which can be scheduled according to a protocol in asymptomatic patients and in patients with worsening clinical status (symptom triggered EMB). In recent years, novel non-invasive tests have been developed such as gene-expression profiling and donor-derived cell-free DNA for screening in stable patients [40, 41]. Nevertheless, the limited access to these tests together with a low accuracy in the early period following HTx has limited their clinical use. The reduction in rsEMB has resulted from the recognition that the occurrence of clinically relevant cardiac rejection is very uncommon in the absence of symptoms or left ventricular systolic dysfunction [42]. Thus, a revised schedule for HTx rejection surveillance has been recently proposed, suggesting three different rsEMB protocols (low-, moderate- and high-frequencies) [9].

## Future perspectives and conclusion

**Fig. 3:**
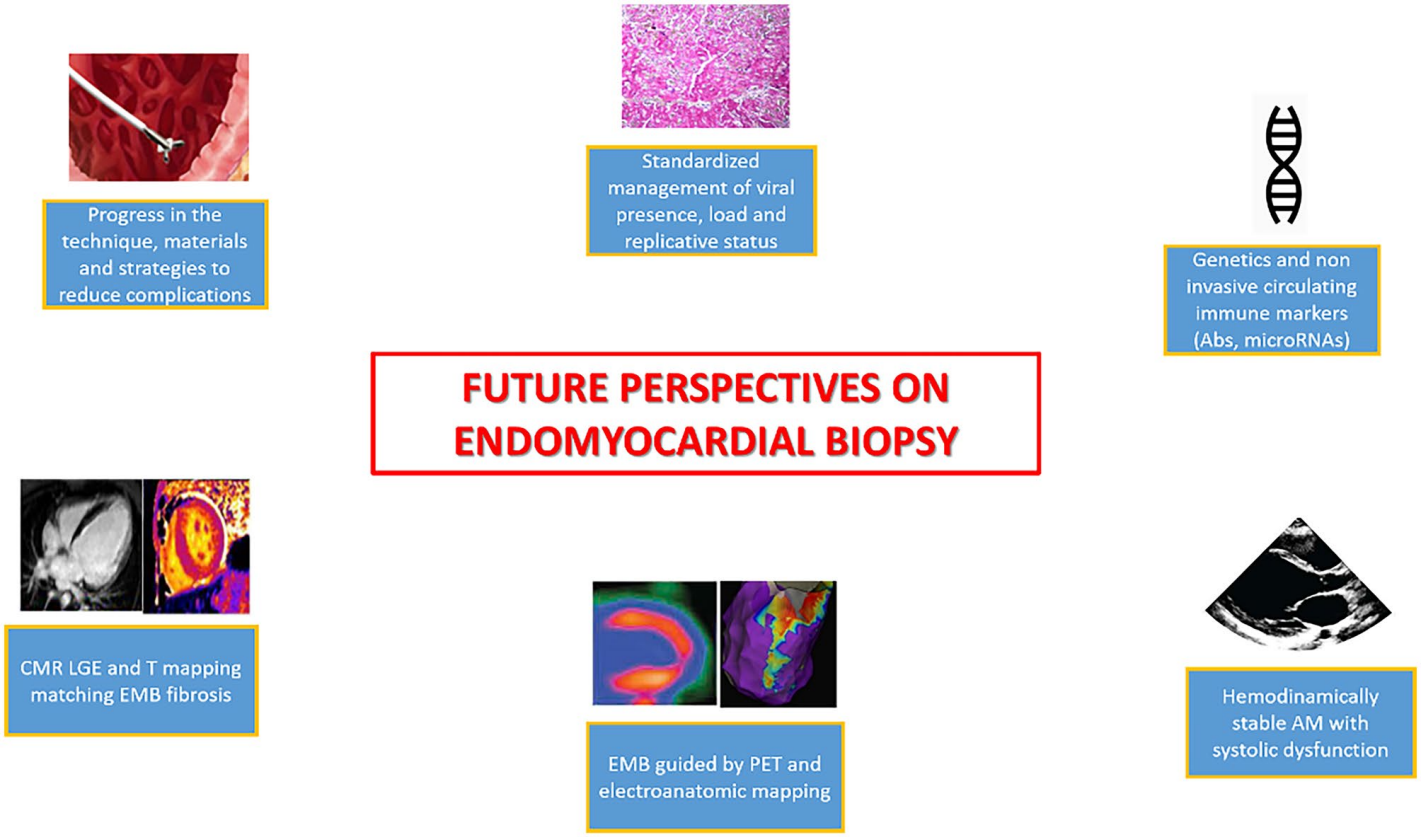
Future perspectives on endomyocardial biopsy. Legend: Ab, antibodies; AM, acute myocarditis; CMR, cardiac magnetic resonance; EMB, endomyocardial biopsy; LGE, late gadolinium enhancement; PET, positron emission tomography

Although a consensus exists about the clinical indications to EMB, this exam gains particular relevance (a) in acute cardiac syndromes, refractory to standard therapies, (b) when non-invasive assessment is not feasible (i.e. CMR not feasible because of frequent arrhythmias, etc..) or yields inconclusive results, (c) for surveillance indications (i.e. reject after HTx) and (d) in selected cases of chronic hemodynamically stable patients with inconclusive non-invasive tests and suspicion of inflammatory disease (i.e. persistently or relapsing increased serum troponin values, ventricular arrhythmias, development of severe LV dysfunction) or underlying cardiomyopathies (i.e. HCM “phenocopies”) (Fig. 3).

Of note, EMB should be performed in centres with specific expertise in evaluating patients with cardiomyopathies and interpreting the immunohistopathological and bio-molecular histological findings. In this perspective, the organization of a “hub-spoke” network should be fully supported in the near future. This approach would allow an accurate selection of best candidates to EMB after consideration of the risk–benefit balance, avoiding taking unnecessary procedural risks. CMR findings such as LGE or increased T values/ECV, the analysis of the genetic background [43] and knowledge of the disease-specific mechanisms of cardiac injury [44] are promising fields of future investigation to refine patients selection.

## Supplementary information

Below is the link to the electronic supplementary material.

Supplementary file1 Video 1 Ventriculography in right anterior oblique projection showing the long sheath over a pigtail catheter in the mid-left ventricular cavity position (AVI 1948 KB)

Supplementary file2 Video 2 Ventriculography in right anterior oblique projection showing the long sheath over a pigtail catheter which is positioned in the right ventricle, in the interventricular septum (AVI 2830 KB)

Supplementary file3 Video 3 Ventriculography in left anterior oblique projection showing the long sheath over a pigtail catheter which is positioned in the right ventricle, in the interventricular septum (AVI 2350 KB)
